# Characterisation of C-C Ligand 7 (CCL7) as Asthma Genetic Marker in Pigtailed Monkey

**DOI:** 10.21315/tlsr2024.35.3.13

**Published:** 2024-10-07

**Authors:** Sela S Mariya, Uus Saepuloh, Novi Febriani, Dyah Perwitasari-Farajallah, Diah Iskandriati, Huda S Darusman, Joko Pamungkas

**Affiliations:** 1 Primate Research Center, IPB University, Jl Lodaya 2 No 5 Bogor, West Java Indonesia; 2 Center for Biomedical Research, National Research, and Innovation Agency of Indonesia, Cibinong-Bogor, West Java, Indonesia; 3 Department of Biology, Faculty of Mathematics and Natural Sciences, IPB University, Jl Agatis, Bogor, Indonesia; 4 Primatology Graduate School of IPB University, Jl Lodaya 2 No. 05, Bogor, Indonesia; 5 School of Veterinary Medicine and Biomedicine IPB University IPB University, Jl Agatis, Bogor, Indonesia

**Keywords:** Asthma, *CCL7* Gene, Pigtailed Monkey, Three-Dimensional CCL7 Protein Structure

## Abstract

The pigtailed monkey (*Macaca nemestrina*) is one of the species that have potency like the cynomolgus monkey that is widely used as an animal model for asthma study. The *CCL7* gene has potential as a genetic marker because of the secreted chemokine that plays a role in asthma. The aims of this research are to characterise the *CCL7* gene of pigtailed monkey, compare the structure of their *CCL7* gene with other primate species and determine model 3D structure protein prediction of CCL7 protein. The amplicons were sequenced, and the results were analysed by the bioinformatics technique. The 3D CCL7 protein structure was predicted using I-TASSER. We have isolated 2221 bp sequences *CCL7* gene and 109 amino acids from pigtailed monkey. Variation of *CCL7* gene sequence between pigtailed monkey and other primate species (*Macaca fascicularis, M. mulatta* and *Homo sapiens*) was found in exon 1, exon 2 and exon 3 as CDS (Coding DNA Sequence) region. The analysis homology of nucleotides and amino acid sequences of the *CCL7* gene indicated that the pigtailed monkey and three other primate species have a high homology rate with an identity score above 90%. Meanwhile, a comparative analysis of CDS and amino acid regions showed that the pigtailed monkey also has the highest similarity with the three other primate species with more than 90% identity score. The 3D structure protein prediction model of the CCL7 pigtailed monkey revealed the highest similarity with *H. sapiens* with an identity value of about 95%. Therefore, the pigtailed monkey *CCL7* gene has high similarity with *H. sapiens*, which means that based on molecular similarity, the pigtailed monkey has the potential to be an animal model for asthma study, especially the study of molecular and the role of CCL7 in asthma pathogenesis.

**Highlights**
- A total of 2221 bp sequences *CCL7* gene and 109 amino acids from pigtailed monkey were have isolated. The sequences of mRNA were deposited to NCBI under accession number OP184473.
- The analysis homology of nucleotides and amino acid sequences of the *CCL7* gene indicated that the pigtailed monkey and three other primate species have a high homology rate with an identity score of more than 90%.
- The 3D structure protein prediction model of the CCL7 pigtailed monkey revealed the highest similarity with *H. sapiens* with an identity value of about 95%.

## INTRODUCTION

The role of animal models in biomedical research to mimic the human response to diseases (Mukherjee *et al*. 2022). Asthma prevalence increased by 50% every year in worldwide (Holgate *et al*. 2015). It impacts increasing the study of asthma which needs animal models for mimicking the disease. Animal models have been used in asthma research such as rats, dogs, goats and non-human primates including rhesus macaque and cynomolgus monkey (Aun *et al*. 2017; Miller *et al*. 2017). Non-human primate is the ideal animal model because they have a similarity in genetics, physiology and anatomy to human (Miller *et al*. 2017; Mariya *et al*. 2019). Human lung has similar anatomy, histology, ultrastructure and size to non-human primate. This is probably to measure the function of lung and bronchoscopy as a detailed analysis of asthma response compared with other animal models of asthma (Plopper & Hyde 2008).

One of the proteins that has been identified that plays role in human asthma reaction is C-C ligand 7 (CCL7). Protein CCL7 is known as *Monocyte Chemotactic Protein-3* (MCP-3) while secreted during inflammation (Opdenakker *et al*. 1993). This protein has the function to recruit the lymphocyte during the inflammation process. The function of protein CCL7 is signalling through lymphocyte cell recruitment. *CCL7* gene is located on the chromosome region 17q11.2–q12 with a total of ~3000 bp of genes consists of 3 exon regions and 2 intron regions (Opdenakker *et al*. 1993; Park *et al*. 2005; Mariya *et al*. 2019; 2020). Asthma research has previously been carried out using cynomolgus monkey as an animal model (Pritchard *et al*. 1983; Plopper & Hyde 2008; Vallender & Miller 2013). The *CCL7* gene of cynomolgus monkey has been characterised. Cynomolgus *CCL7* gene consists of a promotor, 3 exons and 2 intron regions that 97% similar to humans (Mariya *et al*. 2019).

The pigtailed monkey has the potency as an animal model for the disease. In the previous study, the pigtailed monkey has been used as an animal model in biomedical research such as the model of study the novel coronavirus SARS-CoV-2, the causative agent of COVID-19 disease (Melton *et al*. 2021), and research on human immunodeficiency virus (Hatziioannou *et al*. 2015). In this study, we analyse the CCL7 sequences in a pigtailed monkey like we do in the cynomolgus monkey as an animal model for asthma. Perhaps a genetically pigtailed monkey can be used as an animal model of asthma. It can be an alternative animal model of asthma. Identification of one of the genetic markers of asthma such *CCL7* gene in pigtailed monkeys is needed as a first step to use pigtailed monkeys as an animal model of asthma. A previous study report by Mariya *et al*. (2019) found that the *CCL7* gene has potential as a genetic marker of asthma. The aim of this study was to determine the characterisation of the *CCL7* gene as a genetic marker of asthma in pigtailed monkeys. In addition, the secondary objective in this study was to characterise the structure of the *CCL7* gene, compare the structure of the *CCL7* gene with other primates, and predict the three-dimensional structure of the CCL7 protein in the pigtailed monkey.

## METHODS

### Sample Collection

We used archived Blood samples collected from pigtailed monkey. Samples collected in Primate Research Centre of the Institut Pertanian Bogor (IPB) University following approval from the Institutional Animal Care and Use Committee (IACUC).

### Isolation DNA Sequences of the *CCL7* Gene Pigtailed Monkey

**Figure 1:**
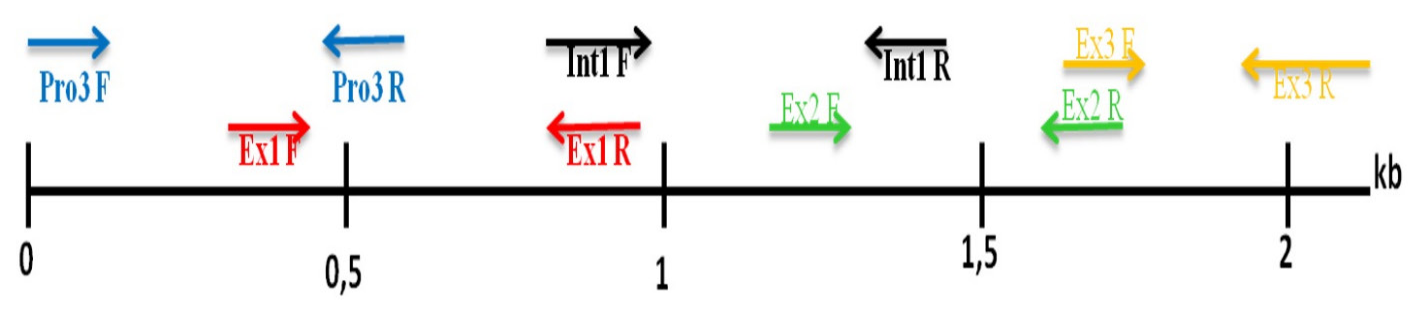
Primer region for amplification *CCL7* gene in pigtailed monkey.

DNA was extracted from pigtailed monkey blood samples using QiaAmp™ DNA blood minimum (Qiagen, Hilden, Germany) according to the instructions company. Amplification was carried out using the Polymerase Chain Reaction (PCR) technique. Amplification of the *CCL7* gene used 5 pairs of forward and reverse primers refers to research by Park *et al*. (2005) with modification by Mariya *et al*. (2019) (Table 1). Primer regions to amplified *CCL7* gene in pigtailed monkey were presented in Fig. 1. Total reagents used in the process PCR is 25 μL consisting of 8 μL nuclease free water, 1 μL [10 pmol/μL] forward primer, 1 μL [10 pmol/μL] reverse primer, 12.5 μL KAPA Hotstart Mastermix and 2.5 μL of template DNA. The PCR technique is performed in Thermocycler Apply Biosystem Perkin Elmer 9700 with the programme pre-denaturation at 94°C for 5 min, denaturation at 94°C for 30 sec, and annealing based on primer sequences used in this research (Table 1) for 30 sec, extension at 72°C for 30 sec and post PCR at 72°C for 7 min. The cycle from denaturation to extension repeats up to 40.

The PCR results were visualised by electrophoresis technique in agarose gel 1.8% and buffer Tris Acetic EDTA (TAE) 1× with SYBR safe [10 mg/mL] as fluorescence. Electrophoresis was run at a voltage of 100 volts for 45 min. The electropherogram was read under UV Gel Doc 2000 with Quantity One (Bio-Rad, USA) programme. The band size is calculated using a 100 pb DNA ladder (Vivantis, Malaysia).

### Bioinformatics Analysis of the *CCL7* Gene

The amplicon 613 bp for promoter 3; 640 bp for exon 1; 628 for intron 1; 530 for intron 2 and 534 for exon 3 region was sent to the 1st Base Malaysia (Selangor, Malaysia) for sequencing. The results were analysed using bioinformatics including variations of nucleotides, homology, comparison of CDS areas, and prediction of three-dimensional structures of CCL7 protein. The sequences were analysed using the Bioedit programme (Ibis Bioscience, USA) and aligned using the programme MEGA7. The sequence resulting from the alignment was then analysed using BLAST (Basic Local Alignment Search Tool) programme from NCBI (National Centre for Biotechnology Information).

Analysis of homology was performed using the NCBI BLAST programme. Characterisation for CDS areas (DNA coding sequences) was carried out using the BLAST programme NCBI. The CDS region of the *CCL7* gene in pigtailed monkey was translated to amino acid sequences using MEGA7 programme. The amino acid translation is also performed using the ORF Finder programme ( http://www.ncbi.nlm.nih.gov/orffinder/). Nucleotide sequences, CDS regions and the amino acid of the CCL7 gene of pigtailed monkey were aligned with other primate species using accession numbers as presented in Table 2.

### Prediction of the Three-Dimensional Structure of the CCL7 Protein Model

A three-dimensional structure prediction protein model was carried out using the I-TASSER programme (http://zhang.bioinformatics.ku.edu/I-TASSER). The I-TASSER programme was used to determine the three-dimensional structure of a query protein obtained by matching it to the structure and function of those proteins is in the Protein Data Bank (PDB). The analog function of global search results is sorted based on the sustainable structure pattern (conserved structure pattern) which exists in the model and is measured using a scoring scheme with combined template-modelling (TM-Score), root-mean-square deviation (RMSD), sequence identity and coverage of the structure alignment. Protein models were visualised using the PyMOL programme (http://www.pymol.org), which allows for further evaluation of the CCL7 protein’s structure and function.

## RESULT AND DISCUSSION

### Amplification of *CCL7* Gene in Pigtailed Monkey

**Figure 2:**
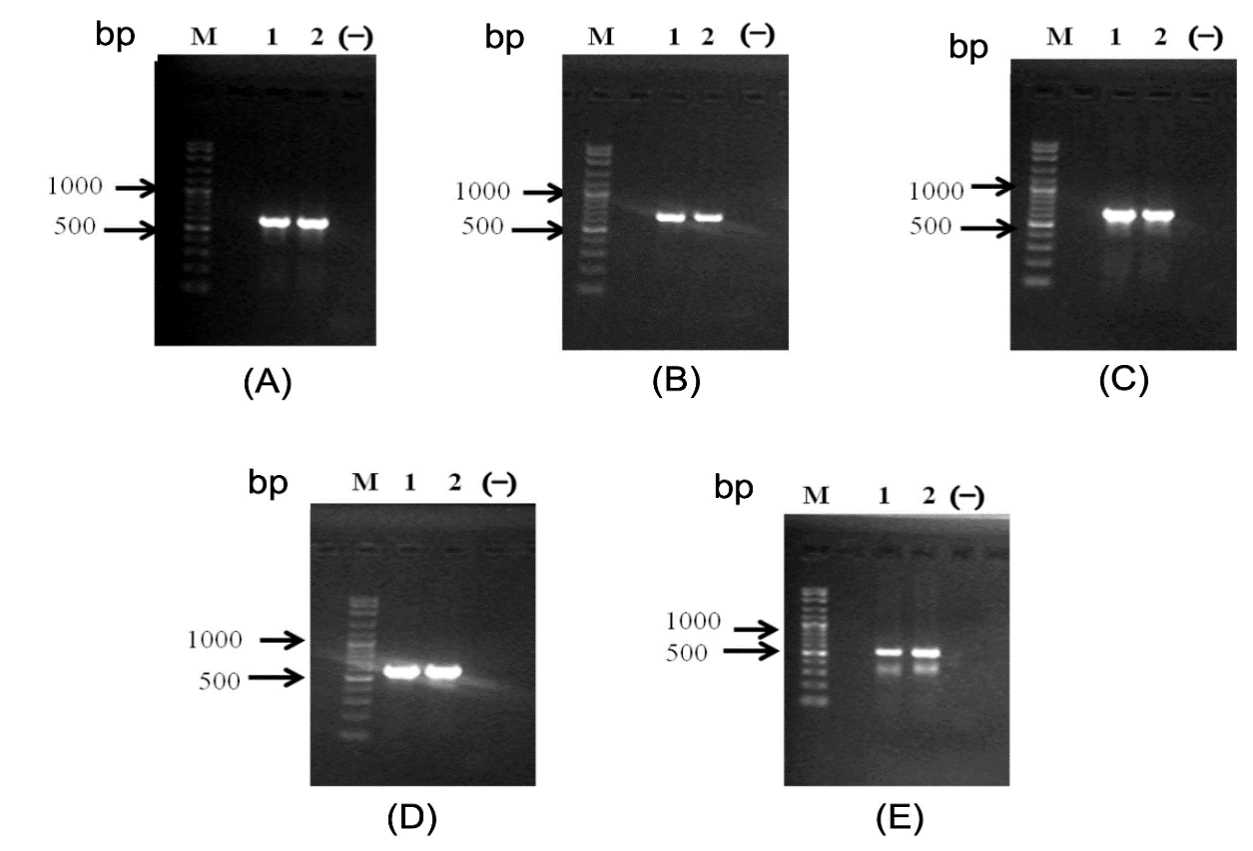
Electropherogram of *CCL7* gene in pigtailed monkey. (A) Promoter 3 (613 bp); (B)Exon 1 (640 bp); (C) Intron 1 (628 bp); (D) Intron 2 and (E) Exon 3 (534 bp). M = Marker 100 bp, 1 and 2 = pigtailed monkey, (−) = negative control.

We used 5 pairs of primers to amplify the *CCL7* gene in pigtailed monkeys; the primer regions are shown in Fig. 2. Annealing positions between the primers overlapped with others. All regions of forward and reverse primer resulted in 2221 bp amplicon sequences of the *CCL7* gene in pigtailed monkey. The prediction of the genetic map has been made based on the *CC7* gene in pigtailed monkey consisting of promoter, exon 1, intron 1, exon 2, intron 2 and exon 3 region.

**Figure 3:**
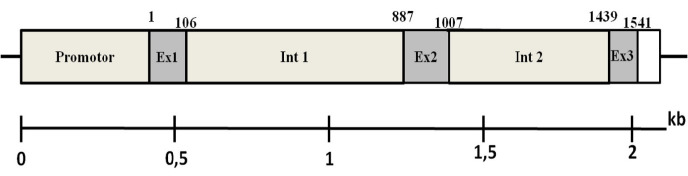
Prediction of a genetic map of *CCL7* gene in pigtailed monkey.

Amplification of the *CCL7* gene in DNA pigtailed monkey has been done using 5 pairs of forward and reverse primers showing single-strand DNA with a variety of amplicon sizes (Fig. 2). The position of primer annealing shows the overlapping area between one primer pair and the others. The merger between forward and reverse primers and overlapping regions of each amplicon resulted in a *CCL7* gene sequence of pigtailed monkey with a size up to 2221 bp. The prediction of the genetic map *CCL7* gene in pigtailed monkey consists of promoter 3, exon 1, intron 1, exon 2, intron 2 and exon 3 region (Fig. 3).

### *CCL7* Gene Sequence Alignment in Pigtailed Monkey

The length of a nucleotide sequence of the *CCL7* gene in pigtailed monkey was 2221 bp and consisted of 3 exons. The molecular weight of exon 1, exon 2 and exon 3 *CCL7* gene in pigtailed monkey consecutively was 105 bp, 120 bp and 103 bp. Our sequences were deposited to GenBank under accession number OP184473. Exon regions in CCL7 pigtailed monkey have been translated to 109 amino acid sequences. Analysis of nucleotide variation was shown a difference between the *CCL7* gene in pigtailed monkey to other primates. Nucleotide variation was found in exon 1, exon 2 and exon 3 (Tables 3–5). Nucleotide variation in *CCL7* gene pigtailed monkey with other primates mainly have been found in exon 1.

The length of exon 1, exon 2 and exon 3 is sequentially 105 bp, 120 bp and 103 bp, respectively. The nucleotide sequence of all three exons translated to 109 an amino acid. Analysis of nucleotide variations showed that there were nucleotide variations between the *CCL7* gene sequence in pigtailed monkey were other primate species, such as *Macaca fascicularis*, *M. mullata* and *Homo sapiens*. The variations were found in 3 exon regions, there are exon 1 (Table 3), exon 2 (Table 4) and exon 3 (Table 5). The number of nucleotide substitution variations of the *CCL7* gene between pigtailed monkey with the three other primate species is more abundant in exon 1 than in exon 2 and exon 3.

### Analysis of Homology Nucleotide and Amino Acids Sequences of the *CCL7* Gene

Analysis of homology of the *CCL7* gene sequences was carried out using BLAST-N and analysis of homology amino acid sequence was performed using BLAST-P (Table 6). The results show the nucleotide sequence and amino acid CCL7 in pigtailed monkey homology with DNA and amino acid sequences CCL7 in other primates. The homology level is based on the E-value BLAST-N which is close to 0 or the identity value which is greater than 70%. Analysis of homology results using BLAST-N, BLAST-X, and BLAST-P indicate an E-value that is close to 0 or a value identity greater than 70% showing that nucleotide sequences are homologous and greater than 25% for analysed amino acid sequence data (Claverie & Notredame 2007).

### The Comparison of CDS Region of the *CCL7* Gene in Pigtailed Monkey

The CDS region of *CCL7* gene in pigtailed monkey was 327 bp. The CDS region is part of a nucleotide sequence consisting of exons that encode a protein. The information regarding the CDS *CCL7* gene in pigtailed monkey is useful for knowing the amino acid and protein structure of the *CCL7* gene that acts as a lymphocyte recruiter inflammation is involved in the pathogenesis of asthma. CDS region of the *CCL7* gene in the pigtailed monkey was compared to the *CCL7* gene of *M. fascicularis, M. mullata* and *H. sapiens* (Table 7). The comparison shows that the pigtailed monkey has the highest similarities with *M. fascicularis* which currently being used as an animal model of asthma (Wang *et al*. 2022).

### Prediction of the 3-Dimensional Structure of the CCL7 Protein Model

A total of 327 bp of the CDS region of the *CCL7* gene were translated to the amino acid. The translation results obtained were 109 amino acids, then predicted the three-dimensional structure of proteins using the I-TASSER programme. I-TASSER is one example of the composite approach and has been ranked as the best method for automated protein structure prediction in the last two CASP experiments (Roy *et al*. 2010). I-TASSER extends its ability and accuracy in modelling large multi-domain protein structures and provides meaningful functional insights for the targets at both the domain- and full-chain levels from the amino acid sequence alone (Zhou *et al*. 2022). The output of the I-TASSER programme is protein structure prediction and functional proteins, such as ligand sites, enzyme active sites and ontology genes. Previous study (Saepuloh *et al*. 2020) predicted the 3D structure of the protein Reverse Transcriptase from Simian Retrovirus Subtype-2 has been successful using I-TASSER programme. TM-score modelling is shown to see the accurate prediction of the protein model (Roy *et al*. 2011).

**Figure 4:**
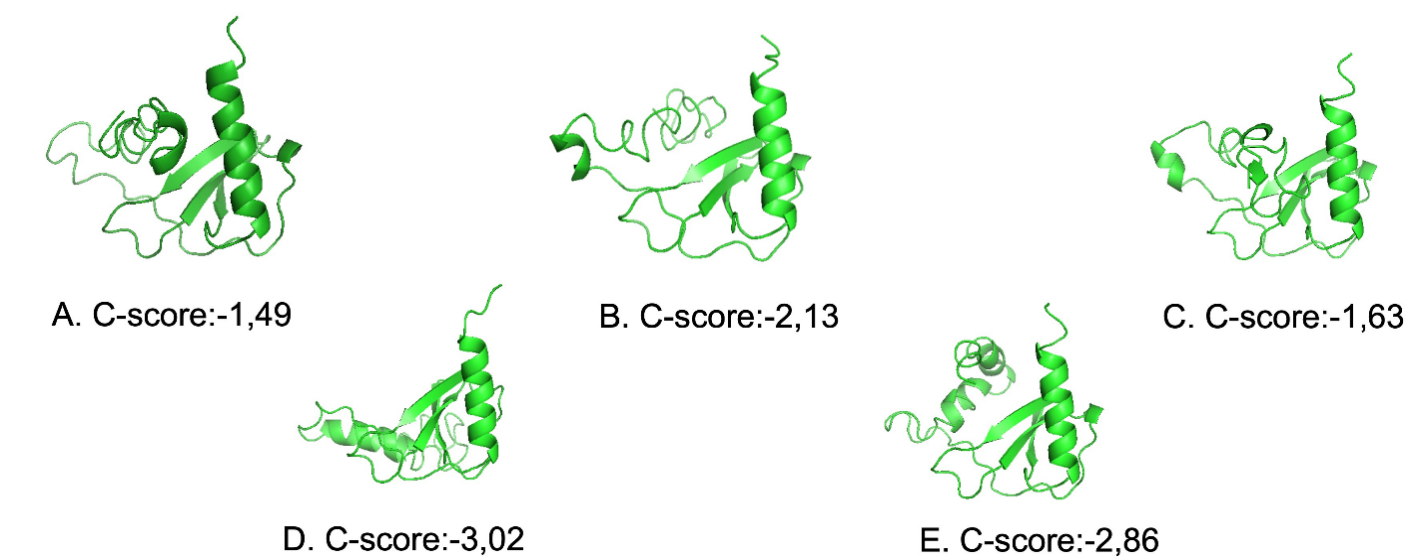
I-TASSER results of structure prediction 3D CCL7 protein in pigtailed monkey. (A) Model 1, (B) Model 2, (C) Model 3, (D) Model 4, and (E) Model 5.

The results of the three-dimensional structure prediction of the pigtailed monkey CCL7 protein are presented in Fig. 4. The prediction of the three-dimensional structure of the CCL7 protein is created using the I-TASSER programme and visualised with the PyMOL programme. Five protein structure models were obtained from the calculation results computations for the pigtailed monkey CCL7 protein. Model 1 was chosen because it has the highest C-score (−1.49). C-score is a value trust whose value ranges from −5 to 2. C-score the highest presented a protein structure model with confidence values the highest. The C-score value correlates with the quality of the resulting model by the I-TASSER programme (Roy *et al*. 2011). In general, structure models with C-score> −1.5 sustain this protein has the appropriate fold chain. Pigtailed monkey CCL7 protein structure model 1 (Fig. 4A) has an accuracy value of TM-score 0.53 ± 0.15 and the estimated RMSD value of 7.2 ± 4.2 Å. RMSD and TM scores were used to measure the topology similarity between the model being sought with the original structure. The TM-score is in the range [0, 1]. A higher value indicates a better structure of protein (Roy *et al*. 2011).

The I-TASSER results showed the structure of the pigtailed monkey CCL7 protein has a high similarity with the structure of the CCL7 protein in *H. sapiens* with an identity value of 95%. These results showed that the prediction for the three-dimensional structure of the pigtailed monkey CCL7 protein obtained was the most suitable structure. The results of the I-TASSER analysis of the pigtailed monkey CCL7 protein showed amino acids as binding sites, such as C44, Y46, R47, F48, I49, K51, K52, I53, P54, Q56, R57, M75, T78, K82, E83, I84 and C85. This result shows that CCL7 is a ligand of a receptor (Ford *et al*. 2019) reported that the CCL7 is a protein as a ligand that binds to three receptors, namely CCR1, CCR2 and CCR3.

This finding is a fundamental study for gene structure characterisation in the pathogenesis of asthma. Asthma is a condition that likely results from complex interactions between multiple genetic and environmental influences (Morales & Duffy 2019). The *CCL7* gene is one of the multiple genes that play role in asthma (Park *et al*. 2005). Gene structures can also change and evolve over time; changing a gene’s coding and noncoding structure can lead to the formation of new genes and neofunctionalisation. In the process of biological development and evolution, organisms have possessed many kinds of regulatory mechanisms to respond to external environmental stimuli (Levy 2019). This mechanism was reported in our previous study by Mariya *et al*. (2020) that have identified a correlation Single Nucleotide Polymorphism (SNP) *CCL7* gene and with asthma response cynomolgus monkey animal model of asthma (Mariya *et al*. 2020). We suggest pigtailed monkeys have the potency to develop animal models of asthma because of their close related to cynomolgus monkeys at the molecular level. Our study reported the *CCL7* gene in pigtailed monkeys for the first time, providing a theoretical basis for the detailed study of *CCL7* genes in the future, especially the function of *CCL7* genes in asthma pathogenesis.

## CONCLUSION

We have isolated 1221 bp of the *CCL7* gene sequence of pigtailed monkey from Indonesia origin. The coding sequences consist of 3 exons, namely exon 1, exon 2 and exon 3 with consecutive sizes of 105 bp, 120 bp and 103 bp and translated to 109 amino acids. The CCL7 amino acid of pigtailed monkey has the highest similarity with *M. fascicularis* and *H. sapiens*. The three-dimensional structure of the CCL7 protein of pigtailed monkey has a high similarity to *H. sapiens*. Like the cynomolgus monkey, this similarity suggests pigtailed monkey has the potency of a translational model to study asthma, especially in the context of molecular studies and the role of CCL7 in asthma pathogenesis.

## Figures and Tables

**Table 1:** Primer used for amplification *CCL7* gene in pigtailed monkey.

No	Primer	Nucleotide sequences (Forward; Reverse)	Temperature annealing (°C)	Primer position (bp)
1	MCP-3 Pro3	F: TGGGTTTTAGAAAGCCACCAGG R: GGGCAATGTGCTTCAAGGAGAA	58	1–614
2	MCP-3 Ex1	F: AAGTGCACCGGCTCAGCAGATT R: TGCAGAGCTGCTGTTTCTGGAA	60	343–983
3	MCP-3 Int 1	F: AGCAGAGTTTGGGATCGGGTGA R: ACACATTACAGCTTCCGGGGA	58	849–1478
4	MCP-3 Ex 2	F: CAAAGACACCGGACTTGGGACG R: ATGCCTCAGGGATGGAGGAGGA	60	1214–1798
5	MCP-3 Ex 3	F: AGACTTCAGTTCTTTTATCCTGG R: TCCACCAAAATCCATGGAAGA	53	1682–2216

*Note*: F = forward, R = reverse

**Table 2:** Nucleotide accession number that is used in single nucleotide polymorphism and homology analysis of nucleotide and amino acid CCL7 sequence.

No	Species	Nucleotide accession number	Amino acid accession number	References
1	*M.fascicularis*	MF06250	AUX81355.1	NCBI
2	M.mulata	AC197944.3	XP_001113381.1	NCBI
3	*H.sapiens*	X72309.1	CAA50406.1	NCBI

**Table 3:** Substitution nucleotide variation to exon 1 *CCL7* gene between pigtailed monkey, cynomolgus monkey and human.

No	Species	Nucleotide position												Variations value

		4	4	5	5	5	5	5	5	5	5	5	5	
		8	9	1	2	2	3	3	5	5	6	8	8	
		9	1	8	4	5	3	7	2	4	5	0	3	
1	*M.nemestrina*	T	G	G	A	T	A	C	T	G	C	T	C	0
2	*M.fascicularis*	-	-	-	-	-	G	-	C	-	-	-	-	2
3	*M.mulata*	A	-	-	-	C	G	-	-	-	-	-	-	3
4	*H.sapiens*	-	T	A	G	C	G	T	-	A	T	G	T	10

**Table 4:** Substitution nucleotide variation to exon 2 *CCL7* gene between pigtailed monkey, cynomolgus monkey and Human.

No.	Species	Nucleotide position					Variations value

		1379	1434	1463	1470	1484	
1	*M.nemestrina*	A	G	T	C	G	0
2	*M.fascicularis*	-	-	-	-	-	0
3	*M.mulata*	-	-	-	-	A	1
4	*H.sapiens*	T	T	C	T	-	4

**Table 5:** Substitution nucleotide variation to exon 3 *CCL7* gene between pigtailed monkey, cynomolgus monkey and human.

No	Species	Nucleotide position		Variations value

		1993	2020	
1	*M.nemestrina*	A	T	0
2	*M.fascicularis*	-	-	0
3	*M.mulata*	-	-	0
4	*H.sapiens*	C	C	2

**Table 6:** Result of BLAST-N (nucleotide) and BLAST-P (amino acid) *CCL7* gene analysis between pigtailed monkey, *M. fascicularis*, *M. mulata* and *H. sapiens*.

Species	*M. nemestrina*			*M. fascicularis*			*M. mulata*			*H. sapiens*		

	QC (%)	E-value	Ident (%)	QC (%)	E-value	Ident (%)	QC (%)	E-value	Ident (%)	QC (%)	E-value	Ident (%)
*M*. *nemestrina*				100	1.00E-73	98	96	2.00E-69	96	96	4.00E-56	91
*M*. *fascicularis*	99	0:00	99				100	7.00E-69	96	96	6.00E-56	91
*M. mulata*	99	0:00	99	98	0:00	99				100	3.00E-56	92
*H. sapiens*	99	0:00	92	98	0:00	91	100	0:00	91			

**Table 7:** The comparison of CDS region between pigtailed monkey, *M. fascicularis, M. mulata* and *H. sapien*.

Species	*M. nemestrina*			*M. fascicularis*			*M. mulata*			*H. sapiens*		

	QC (%)	E-value	Ident (%)	QC (%)	E-value	Ident (%)	QC (%)	E-value	Ident	QC (%)	E-value	Ident
*M*. *nemestrina*												
*M*. *fascicularis*	100	9.00E-166	99									
*M.mulata*	99	2.00E-161	99	99	5.00E-163	99						
*H. sapiens*	100	9.00E-141	95	100	2.00E-142	95	100	9.00E-141	96			
